# Polymer-Based Artificial Solid Electrolyte Interphase Layers for Li- and Zn-Metal Anodes: From Molecular Engineering to Operando Visualization

**DOI:** 10.3390/polym17222999

**Published:** 2025-11-11

**Authors:** Jae-Hee Han, Joonho Bae

**Affiliations:** 1 Department of Materials Science and Engineering, Gachon University, Seongnam 13120, Republic of Korea; jhhan388@gachon.ac.kr; 2 College of Physics and Semiconductor Science, Gachon University, Seongnam 13120, Republic of Korea

**Keywords:** artificial solid electrolyte interphase, polymer-based interphase, lithium metal anode, aqueous zinc metal anodes, operando characterization

## Abstract

Metal anodes promise improvements in energy density and cost; however, their performance is determined within the first several nanometers at the interface. This review reports on how polymer-based artificial solid electrolyte interphases (SEIs) are engineered to stabilize Li and aqueous-Zn anodes, and how these designs are now evaluated against operando readouts rather than post-mortem snapshots. We group the related molecular strategies into three classes: (i) side-chain/ionomer chemistry (salt-philic, fluorinated, zwitterionic) to increase cation selectivity and manage local solvation; (ii) dynamic or covalently cross-linked networks to absorb microcracks and maintain coverage during plating/stripping; and (iii) polymer–ceramic hybrids that balance modulus, wetting, and ionic transport characteristics. We then benchmark these choices against metal-specific constraints—high reductive potential and inactive Li accumulation for Li, and pH, water activity, corrosion, and hydrogen evolution reaction (HER) for Zn—showing why a universal preparation method is unlikely. A central element is a system of design parameters and operando metrics that links material parameters to readouts collected under bias, including the nucleation overpotential (*η_nuc_*), interfacial impedance (charge transfer resistance (*R_ct_*)/SEI resistance (*R_SEI_*)), morphology/roughness statistics from liquid-cell or cryogenic electron microscopy (Cryo-EM), stack swelling, and (for Li) inactive-Li inventory. By contrast, planar plating/stripping and HER suppression are primary success metrics for Zn. Finally, we outline parameters affecting these systems, including the use of lean electrolytes, the N/P ratio, high areal capacity/current density, and pouch-cell pressure uniformity, and discuss closed-loop workflows that couple molecular design with multimodal operando diagnostics. In this view, polymer artificial SEIs evolve from curated “recipes” into predictive, transferable interfaces, paving a path from coin-cell to prototype-level Li- and Zn-metal batteries.

## 1. Introduction

Rechargeable batteries that deploy metal anodes—most prominently Li and Zn—promise advancements in energy density and cost, thereby broadening their potential use from mobile systems to stationary storage applications [1,2]. Li combines an ultrahigh theoretical capacity (3860 mAh g^−1^) with the lowest redox potential among metals, making it a benchmark anode for next-generation high-energy chemistries [1,3]. In addition, Zn is compelling for aqueous systems owing to its elemental abundance, intrinsic safety, and compatibility with low-cost electrolytes, making it a pragmatic solution for grid-scale storage [4,5]. However, these advantages are consistently undercut by interfacial instabilities, such as morphological roughening and dendrites, parasitic reactions, and Coulombic efficiency loss, which are rooted in the chemistry and mechanics of the solid electrolyte interphase (SEI) that forms at the metal/electrolyte boundary [6,7].

The SEI originates from electrolyte reduction on highly reducing metal surfaces, yielding a nanometric, ion-conducting, electron-insulating film that throttles further reactions [6]. However, native SEIs on Li and Zn are typically heterogeneous, mechanically fragile, and dynamically rebuilt during cycling; cracking and dissolution expose fresh metal, accelerate electrolyte consumption, and enable filamentary growth and short circuits [8,9]. In aqueous Zn cells, hydrogen evolution and passivation (e.g., ZnO/Zn(OH)_2_) further complicate SEI function and plating uniformity [5]. These observations motivate a shift from relying on “self-formed” interphases to deliberately engineered interfaces that integrate ionic transport, chemical stability, and mechanical robustness [7,10].

Artificial SEIs based on polymers have therefore emerged as versatile platforms for stabilizing Li and Zn anodes. Through molecular design involving the tuning of their segmental mobility, dielectric constant, coordination chemistry, cross-link density, and interfacial energy, polymeric layers can homogenize ion flux, regulate solvation/desolvation, and impart mechanical resistance to protrusion growth [11,12,13]. Representative strategies include (i) ion-conductive matrices (e.g., polyethers, polyacrylonitrile (PAN)-based, and zwitterionic polymers), (ii) hybrid organic/inorganic composites, and (iii) reactive/interpenetrating interphases that evolve into LiF/Li_2_O-rich chemistries under bias [11,14,15]. For aqueous Zn, polymeric and zwitterionic coatings can suppress corrosion and hydrogen evolution while guiding planar Zn deposition, thereby extending symmetric cell lifetimes [16,17]. Despite rapid progress, an integrative framework linking molecular structure and mechanics to interfacial transport, electrochemistry, and failure statistics across both Li and Zn platforms has not yet been developed [18]. A second gap lies in the transition from post-mortem characterization to true operando visualization. Advances in in situ/operando transmission electron microscope (TEM) and spectroscopy have led to the identification of SEI nucleation, densification, and fracture, and metal plating pathways under realistic polarization [19,20]. For Li, cryo-EM and low-dose cryogenic electron tomography (cryo-ET) can resolve mosaic versus multilayer SEIs, and a LiF-rich nanocrystalline framework can guide subsequent Li regrowth [21,22,23]. For Zn, ordered planar plating/stripping under operando conditions enables deep cycling behavior [24]. However, integrative reviews that explicitly map polymer interphase design rules to operando metrics for both Li and Zn remain limited [11,18,19].

Herein, we address these gaps by synthesizing design principles for polymer-based artificial SEIs with state-of-the-art operando visualization across Li and Zn systems. We aim to (i) delineate the physical chemistry governing interphase formation and evolution; (ii) map molecular handles in polymers to interfacial transport, mechanics, and electrochemistry; (iii) highlight metal-specific contrasts (organic versus aqueous environments); (iv) curate operando toolkits and the interphase observables they access; and (v) articulate a forward-looking, metrics-anchored design paradigm that can translate benchtop demonstrations into commercially relevant devices [1,4,7]. By coupling molecular engineering with visualization-driven validation, we aim to establish actionable rules for realizing durable, dendrite-free metal anodes.

## 2. Fundamentals of SEI Layers on Metal Anodes

The SEI layer greatly affects the characteristics of metal-anode batteries. Originating from electrolyte reduction during initial formation, the SEI passivates the metal surface while allowing cation transport, and this idea stemmed from Peled’s seminal model and has since evolved [6,25]. The early decomposition of Li yields a hybrid inorganic/organic nanocomposite that typically includes LiF, Li_2_CO_3_, and lithium alkyl carbonates embedded in an amorphous matrix; the precise phase distribution depends on the salt/solvent and current history [25]. For Zn in aqueous media, the interphase chemistry is even more multicomponent: ZnO/Zn(OH)_2_, basic zinc salts (e.g., basic sulfate/carbonate), phosphates, and hydrated organic moieties can all appear, with the composition strongly dictated by the pH, anions, and additives [26,27]. Functionally, an effective SEI must suppress electron tunneling while providing a low-impedance pathway for Li^+^ or Zn^2+^. Their mechanical attributes are equally important; the layer must resist fracture and maintain contact as the metal breathes during plating/stripping. Theoretical and experimental studies have shown that mechanical stability (elastic modulus, toughness, and adhesiveness) governs whether protrusions are amplified or self-limiting, whereas in polymeric environments, a separator/SEI with a modulus exceeding that of Li can suppress interfacial roughening [28,29].

Regarding the mechanics, there have been numerous studies available in the literature. Artificial (polymer or hybrid) SEI layers must balance two seemingly conflicting mechanical requirements: high stiffness to resist dendrite penetration and sufficient toughness or flexibility to maintain interfacial contact during lithium’s volume changes [30,31]. A very rigid coating (high elastic modulus) can suppress Li dendrite growth by physically blocking or deflecting protrusions, but overly stiff or brittle layers risk cracking or delaminating as the Li metal expands and contracts. Conversely, a very soft or low-modulus polymer conforms easily to the anode and accommodates volume changes, yet it may lack the strength to hinder dendrite advancement [30]. Therefore, an ideal polymer SEI is mechanically robust (to prevent dendrites) while also elastic/adhesive enough to remain intact on the electrode through repeated cycling. This challenge has been acknowledged in recent studies, and researchers have developed material designs to navigate the stiffness versus flexibility trade-off rather than simply tolerating it [30]. For example, Wang et al. [30] demonstrated that a hybrid solid electrolyte with a rigid Li_1.5_Al_0.5_Ge_1.5_(PO_4_)_3_ (LAGP) core and a flexible poly(vinylidene fluoride) (PVDF)- hexafluoropropylene (HFP) shell achieved both high modulus (≈25 GPa) and conformal interfacial contact, successfully suppressing dendrite formation during cycling. Similarly, Youk et al. [31] reviewed polymeric coatings such as Li polyacrylic acid (LiPAA) and poly(dimethylsiloxane) (PDMS) that exhibit high elasticity and moderate modulus, enabling self-adaptive interfacial behavior to accommodate Li deformation without sacrificing structural integrity. These examples illustrate concrete polymer strategies that reconcile the contradictory requirements of rigidity and flexibility in artificial SEI design.

The type of electrolyte affects both the first-cycle SEI chemistry and its repair kinetics. In organic electrolytes (typical for Li), salt anion selection and solvent reduction routes control LiF-rich versus carbonate-rich mosaics; in aqueous Zn systems, water activity, complexation, and buffering govern competition among dendrite growth, corrosion, and hydrogen evolution [4,25,27]. Notably, several water-lean/additive strategies can promote thin ionomer-like interphases and the partial self-healing of Zn, thus improving reversibility [32]. Defects, including cracks, voids, and compositional inhomogeneity, localize the current and seed filamentary growth across both chemistries. This linkage between SEI integrity and dendrites has been documented through modeling and experiments and is now a common design target for interphase engineering [26,33]. Historically, the SEI structure has been analyzed via ex situ images (X-ray photoelectron spectroscopy (XPS), time-of-flight secondary ion mass spectrometry (ToF-SIMS), and TEM). New operando microscopy and spectroscopy techniques can now resolve how the SEI nucleates, thickens, and is reconstructed under bias. In situ/operando liquid-cell TEM can visualize lithium deposition and the stepwise build-up of mosaic SEIs, while operando spectrum imaging (e.g., electron energy loss spectroscopy (EELS)) and multimodal X-ray/electron probes can quantify the concurrent chemistry and morphology [20,34,35]. These tools enable direct evaluation of transport/mechanics hypotheses that previously relied on indirect inference.

Taken together, the chemistry, architecture, and mechanics of the SEI control nearly every macroscale performance metric, including the Coulombic efficiency, rate capability, and safety. Mastering these fundamentals is a prerequisite for rational interface design, including polymer-based artificial SEIs that deliberately tune the ion transport, elastic modulus, and interfacial energy of Li and Zn [11,36].

## 3. Molecular Engineering of Polymer-Based Artificial SEI Layers

**Figure 1:**
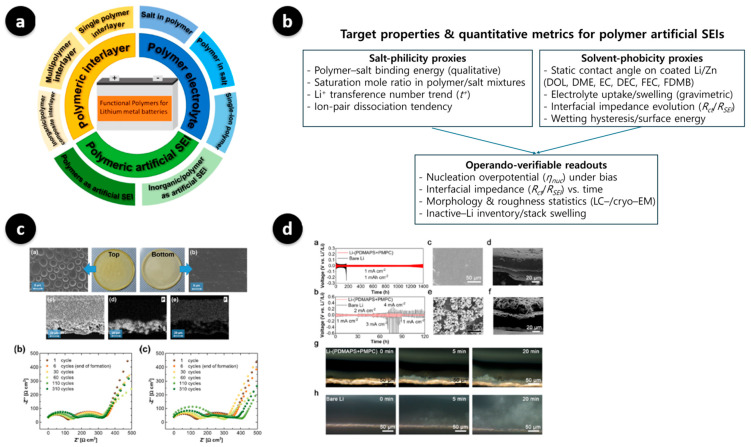
(**a**) Functional polymer classes relevant to Li-metal anodes (polymer artificial SEI/interlayer/electrolyte), shown as a design map for interphase engineering. Reproduced from Ma et al., Polymers (2022) [36] under the Creative Commons CC BY 4.0 license. (**b**) Target properties and quantitative metrics (author-generated schematic). (**Left**): *salt-philicity* screening proxies (qualitative polymer–salt binding, saturation molar ratio in polymer/salt mixtures, Li^+^ transference number trend, ion-pair dissociation tendency). (**Right**): *solvent-phobicity* proxies on coated Li/Zn (static contact angle in representative solvents/electrolytes such as 1,3-dioxolane (DOL), 1,2-dimethoxyethane (DME), ethylene carbonate (EC), diethyl carbonate (DEC), fluoroethylene carbonate (FEC), 1-fluoro-3,5-dimethoxybenzene (FDMB); electrolyte uptake/swelling; interfacial impedance evolution of *R_ct_*/*R_SEI_*; wetting hysteresis/surface energy). (**Bottom**): *operando-verifiable readouts* that link materials design to device behavior (nucleation overpotential *η_nuc_*, temporal trends of *R_ct_*/*R_SEI_*, morphology/roughness statistics from liquid cell (LC)-/cryo-EM, inactive-Li inventory/stack swelling). This panel is an author-drawn synthesis inspired by Ref. [37] and related literature; it is not a reproduction of any published figure. (**c**) Ceramic-in-polymer hybrid interphase [38]: SEM image of the top and bottom membrane surfaces with photographs, cross-section SEM image, EDX maps of P and F, and Nyquist plots recorded at selected cycles, showing lower interfacial/charge-transfer impedance and a more benign evolution for the coated system than for the non-coated control. Adapted from Overhoff et al., *ACS Applied Materials & Interfaces* (2022) [38], open-access under the Creative Commons Attribution 4.0 license. (**d**) Zwitterionic polymer artificial SEI on Li (Li-(poly(3-dimethyl(methacryloyloxyethyl)ammonium propane sulfonate) (PDMAPS)+poly(2-methacryloyloxyethyl phosphorylcholine) (PMPC))): (**a**,**b**) long-term galvanostatic cycling and current-step response in Li‖Li symmetric cells [38]; (**c**–**f**) plan-view and cross-section SEM image after cycling; (**g**,**h**) in situ optical microscopy images obtained during plating, all showing smoother interfaces and suppressed protrusions relative to bare Li [39]. Adapted from Jin et al., *ACS Applied Materials & Interfaces* 2021, with permission from the American Chemical Society [39]. Note: Lower-case letters visible inside some sub-images correspond to the original figures’ sub-panel labels and are retained for traceability; scale bars and authors’ original annotations are preserved.

Building on these principles, molecular engineering of polymer-based artificial SEI layers focuses on tuning polymer chemistry at the atomic scale to control ion transport and interfacial stability. By adjusting backbone polarity, side-chain functionality, and crosslinking density, researchers can optimize Li^+^ coordination and suppress parasitic reactions at the metal interface. In this section, we will discuss how molecular engineering of polymer-based artificial SEI layers has recently been reported. Figure 1 summarizes how polymer chemistry maps to the interfacial function and operando outcomes. Figure 1a organizes the working space into polymeric artificial SEIs, polymer interlayers, and polymer electrolytes, highlighting the subfamilies referenced throughout this review [36]. Figure 1b shows a schematic that groups the quantitative design metrics used in this field: qualitative polymer–salt binding and the saturation mole ratio together with the Li^+^ transference-number trend and ion-pair dissociation as *salt-philicity* proxies, and the static contact angles in representative solvents/electrolytes, electrolyte uptake/swelling, and the evolution of interfacial impedance *R_ct_*/*R_SEI_* as *solvent-phobicity* proxies. These metrics motivate *operando-verifiable* readouts, which include the nucleation overpotential *η_nuc_*, temporal trends of *R_ct_*/*R_SEI_*, morphology/roughness statistics from liquid-cell or cryo-ET, and inactive-Li inventory or stack swelling, and reflect the design logic summarized by Huang et al. [37]. Figure 1c presents a polymer–ceramic hybrid coating wherein top/bottom surface and cross-sectional scanning electron microscope (SEM) images and energy dispersive X-ray spectroscopy (EDX) maps reveal the hybrid architecture, and Nyquist plots recorded at selected cycles show a lower interfacial/charge-transfer impedance and a more benign evolution than the uncoated control [38]. Finally, Figure 1d shows a high-dielectric zwitterionic coating on lithium. Long-term galvanostatic and step tests, post-mortem SEM, and in situ optical imaging all indicate smoother plating/stripping and the suppression of protrusions relative to bare lithium [39]. Together, these panels constitute the topics addressed in Section 3, including chemistry classes and architectures (Section 3.1), target properties and quantitative metrics (Section 3.2), and design parameters mapped to operando observables (Section 3.3).

### 3.1. Chemistry Classes and Architectures

The matrices of these systems include poly(ethylene oxide) (PEO), PVDF, ionomer/zwitterionic polymers, PAN/polyamide (PA), block/brush copolymers, polymer-in-salt systems, and polymer–ceramic hybrids (Al_2_O_3_, Li_7_La_3_Zr_2_O_12_ (LLZO)). Representative evidence for and reviews of these systems include [38,39,40,41,42].

The design intent differs by class as zwitterionic/ionomer matrices promote cation-selective transport and suppress solvent cotransport, block/brush architectures decouple the mechanical modulus from segmental motion, and polymer–ceramic hybrids increase the modulus and thermal stability while preserving percolative Li^+^ pathways [40,41]. Overhoff et al. demonstrated that ceramic-in-polymer hybrid electrolytes with functionalized active ceramic fillers, a single-ion conducting polymer matrix, and controlled solvent/swelling agents can yield electrolytes combining good ionic conductivity and reduced solvent uptake, thereby improving safety and stability [38].

### 3.2. Target Properties & Quantitative Metrics

As summarized in Figure 1b and based on Huang et al. [37], we grouped the target properties and their quantitative metrics into three blocks. First, *salt-philicity* is screened using polymer–salt binding trends and the saturation molar ratio in polymer–salt mixtures, along with the qualitative direction of the Li-ion transference number and ion-pair dissociation. Second, *solvent-phobicity* on the coated Li or Zn is captured via static contact angles in representative electrolytes, electrolyte uptake or swelling, and the evolution of the interfacial impedance (*R_ct_* or *R_SEI_*). Third, we connect these material metrics to operando-verifiable readouts, including the nucleation overpotential, temporal trends of *R_ct_* or *R_SEI_*, morphology and roughness statistics from LC or cryo-EM, and the inventory of inactive lithium or stack swelling. The following framework defines how the chemistry and architecture choices outlined in Section 3.1 are translated into measurable interfacial functions:

Transport: Bulk/interfacial ionic conductivity *σ* (S cm^−1^, 25 °C), cation transference number *t^+^* (Li^+^/Zn^2+^), and interfacial charge-transfer resistance *R_ct_*/*R_SEI_* (electrochemical impedance spectroscopy (EIS)).

Mechanical properties and adhesion: Elastic modulus *E*, hardness *H*, critical fracture energy *G_c_* (nanoindentation/AFM/peel), and wetting/contact angle on Li or Zn.

Electrochemical performance proxies: Nucleation overpotential *η_nuc_*, symmetric-cell lifetime at a specified current density (*J*) (mA cm^−2^) and areal capacity (mAh cm^−2^), and short-term probability under a lean electrolyte.

Aqueous-Zn-specific: Hydrogen evolution current density (chronoamperometry), corrosion rate, and self-discharge.

These metrics are used to normalize previously reported values and connect material design with interfacial function [36,42].

#### Measurement Notes (How to Measure Each Metric)

- *σ* (ionic conductivity): Through-plane EIS using blocking electrodes; normalize by thickness/area; report the temperature (e.g., 25 °C) and humidity for hydrophilic films [36].
- *t^+^* (cation transference number): Bruce–Vincent DC polarization with small-signal EIS before and after correction; state the salt concentration and cell symmetry [36,42].
- *R_ct_*/*R_SEI_*: Extract these parameters from the EIS results using an explicitly defined equivalent circuit; identify the high-frequency semicircle attributed to the interphase; control the contact resistance and temperature [42].
- *E*, *H*, and *G_c_*: Nanoindentation or AFM force–distance mapping are used to determine the elastic modulus (*E*) and hardness (*H*), while peel or double-cantilever beam (DCB) tests quantify the adhesion strength and critical fracture energy (*G_c_*) [36].
- *η_nuc_* (nucleation overpotential): The potential dip at the onset of galvanostatic deposition is recorded; report the current density, electrolyte, and rest history [42].
- Symmetric-cell lifetime: Specify *J* (mA cm^−2^), the areal capacity per cycle (mAh cm^−2^), stack pressure, and electrolyte/negative-to-positive (N/P) ratio [36].
- Aqueous-Zn metrics: Determine the HER current via chronoamperometry versus reversible hydrogen electrode (RHE), the corrosion rate by Tafel extrapolation or mass-loss measurements, and self-discharge by open circuit voltage (OCV) decay [36].

### 3.3. Design Parameters and Operando Metrics

Table 1 summarizes key molecular engineering strategies for polymer-based artificial SEI layers, linking design parameters to their targeted interfacial functions and corresponding operando observables. Beyond ensuring simple passivation, polymeric interphases are now expected to deliver multi-functional roles, including ion-selective transport, mechanical homogenization, and dynamic self-healing. These functions can only be validated by advanced operando techniques such as liquid-cell transmission electron microscopy (LC-TEM), operando EIS, liquid cell scanning transmission electron microscopy (LC-STEM), and X-ray characterization.

- Zwitterionic or ionomeric side chains have emerged as effective motifs for promoting cation-selective transport while suppressing solvent co-transport. Such interphases facilitate uniform lithium plating, accompanied by reduced drift in *R_ct_*, as confirmed by LC-TEM and operando EIS measurements [39,41]. These findings highlight the role of molecular dipoles in achieving controlled ion flux across the SEI.
- Fluorinated or salt-philic side chains drive the formation of inorganic-rich, electronically insulating SEI layers, often enriched in LiF. This results in denser mosaic-type morphologies with suppressed porosity growth, as observed in operando LC-STEM and X-ray studies [34,37]. Such design principles leverage the strong interfacial stability of fluorinated chemistries to inhibit uncontrolled dendritic propagation.
- Incorporating ceramic fillers such as Al_2_O_3_ or LLZO into polymer matrices provides enhanced mechanical modulus and enables more homogeneous current distribution. Operando LC-TEM studies have shown that these hybrid systems reduce tip-growth probability and maintain smoother electrodeposition fronts, underscoring the importance of mechanical reinforcement in suppressing localized instabilities [35,38,40].
- Finally, dynamic cross-links or supramolecular bonding motifs impart self-healing capabilities to the artificial SEI. These reversible interactions enable crack recovery and sustain interfacial coverage during extended cycling. Recent advances have further demonstrated that supramolecular interaction frameworks within polymer electrolytes can regulate Li^+^ solvation dynamics and enhance interfacial homogeneity, thereby achieving stable lithium deposition [43]. Correspondingly, operando studies report stable plating morphologies and slower impedance rise when such adaptive networks are employed [36].

In summary, Table 1 illustrates how rational molecular design of polymer-based artificial SEI layers translates into distinct interfacial functions and measurable operando signatures. By correlating design strategies with real-time diagnostic evidence, these studies provide a roadmap for establishing robust and multifunctional artificial SEI layers that can effectively stabilize lithium metal anodes.

## 4. Artificial SEI Layers for Li-Metal Anodes: Recent Advances

The central difficulty at the Li metal surface is the coupling of nucleation–growth asymmetry, creep/porosity evolution, and accumulation of inactive Li. As solvent depletion develops, growth transitions from mossy to tip-driven dendritic modes [44], and electrochemical kinetics co-evolve with surface topography such that pits and dendrites reinforce each other [45]. Consequently, an artificial SEI must simultaneously block electrons, support cation-selective transport, and delay mechanical failure [42].

**Figure 2:**
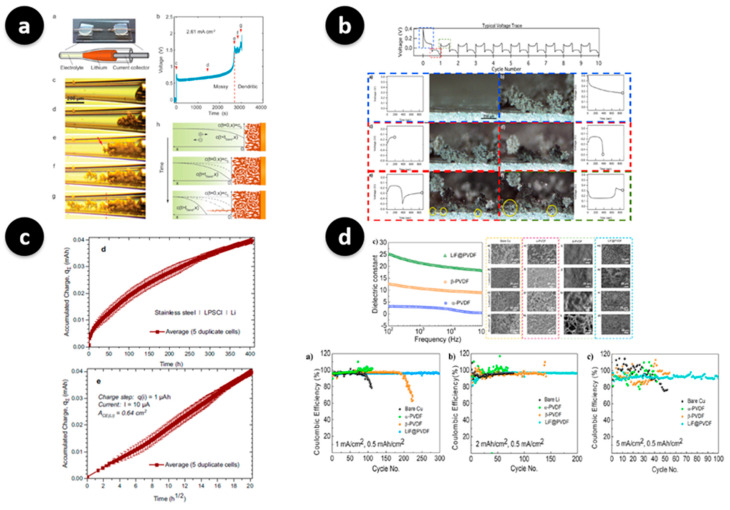
Failure modes at Li-metal interfaces and polymer artificial SEI systems with operando readouts. (**a**) Capillary-cell in situ optics showing the transition from mossy to tip-grown dendritic regimes at Sand’s time, together with a schematic of the concentration-polarization mechanism (source panels from Figure 1a–h). The red dashed line marks the transition boundary between the pre- and post-Sand’s-time Li deposits, and the red arrow indicates the first emergence of a dendrite. Reproduced from Bai et al., *Energy & Environmental Science* (2016) [44] (under the Creative Commons CC BY 4.0 license.). (**b**) Operando video microscopy images showing time-synchronized voltage traces with morphology evolution, including deposition, stripping, pit initiation, and re-nucleation using still frames from the first cycles (source panels from Figure 1). Colored dashed boxes (red/blue) are annotations added in this figure to delineate distinct morphology–voltage segments (e.g., early deposition vs. stripping/pit-initiation). Reproduced from Wood et al., ACS Central Science (2016) [45]. This article is published under the terms of the ACS AuthorChoice License, which permits copying and redistribution of the article or any adaptations for non-commercial purposes. (**c**) Coulometric titration time analysis (CTTA) of SEI growth under sulfide solid electrolyte conditions (stainless steel | LPSCl | Li). The accumulated parasitic charge Q_Σ_ from five duplicate cells is plotted (upper) versus time (source panel from Figure 1d) and (lower) versus t^1/2^ (source panel from Figure 1e), where the linearity in the Q_Σt^1/2^_ plot indicates diffusion-limited t^1/2^ growth kinetics of side reactions/SEI formation. Reproduced from Aktekin et al., Nature Communications (2023) [9] with permission under the Creative Commons Attribution 4.0 International license. (**d**) High-dielectric PVDF-based artificial SEIs: dielectric spectra comparing α-PVDF, β-PVDF, and LiF@PVDF (source panels from Figure 1c), plan-view SEM image of Li deposits at increasing areal capacities for the three coatings (source panels from Figure 2a–p), and representative Coulombic-efficiency benchmarks (source panels from Figure 3a–c). These images show more uniform plating was achieved with LiF@PVDF. Adapted from Tamwattana et al., *ACS Energy Letters* (2021) [14] (permission from the American Chemical Society). Note: Lower-case letters visible inside some sub-images correspond to the original figures’ sub-panel labels and are retained for traceability; scale bars and authors’ original annotations are preserved.

As illustrated in Figure 2a, capillary cell observations capture the transition from compact deposits to tip-driven dendrites as the concentration polarization increases [44]. Figure 2b shows an operando microscopy video that links the pit formation during stripping with the dendritic regrowth during the subsequent plating half-cycle [45]. To quantify the electrochemical cost of these processes, Figure 2c applies coulometric titration time analysis to stainless-steel | LPSCl | Li cells, plotting the accumulated parasitic charge Q_Σ_ from replicate cells versus time and versus t^1/2^; the linear Q_Σt^1/2^_ relation evidences diffusion-limited growth of side reactions/SEI under sulfide solid electrolyte conditions, providing an absolute, kinetics-focused benchmark for interphase design [9]. Finally, Figure 2d shows a material design in which a high-dielectric PVDF-based artificial SEI improves the plating uniformity and Coulombic efficiency at practical areal loadings, as corroborated by the dielectric spectra and plan-view SEM image [14].

Polymer-based artificial SEIs can be organized via three design techniques: (i) side-chain/ionomer chemistry for tuning local solvation and the Li^+^ transference number; (ii) covalent or supramolecular cross-linking for absorbing microcracks; and (iii) polymer–ceramic hybrids for achieving a tradeoff between modulus and ionic transport [40,42,46]. For example, dispersing Al_2_O_3_ or garnet-type LLZO fillers in a PEO matrix increases the shear/crack resistance and organizes interfacial ion pathways, reducing the probability of tip growth [40]. Overhardening, however, compromises wetting and adhesion; therefore, a balance of an intermediate modulus with cation-selective transport and good wetting is required [46]. The second major shift is methodological, emphasizing the establishment of a feedback loop through operando observation. Techniques such as LC- and cryo-EM combined with complementary spectroscopy enable direct correlation of nucleation overpotential, current distribution, and morphology evolution under bias to reveal the chemistry of the interphase. Here, the LiF-rich composition, mosaic density, and pore-growth rate have been identified as important metrics [34,44]. Successful designs for Li systems rarely exhibit a one-to-one relationship with other metals. For instance, Na differs in solvation, interfacial reactivity, and mechanical response, leading to distinct growth modes and SEI chemistries, even with the same coating [47]. Accordingly, this section has focused on Li by outlining the failure modes, detailing the polymer-SEI strategies, and finally, indicating the operando metrics used to verify them.

This gap between laboratory demonstrations and practical cell operation is well defined. Under lean electrolytes, a limited N/P ratio, and high areal capacity/current density, coatings must restrain cell swelling and inactive-Li accumulation [48,49]. They also need to be co-designed with a liquid electrolyte, which is the dominant bottleneck in current Li metal batteries (LMBs) [50]. At the pouch-cell scale, uniform stack pressure, robust current-collector contact, and controlled electrolyte stoichiometry must be reproducible [51]. Therefore, mapping polymer composition/architecture to operando metrics, including *η_nuc_*, *R_ct_*/*R_SEI_*, morphological roughness, stack swelling, and inactive-Li, provides a pathway toward commercialization.

## 5. Artificial SEI Layers for Zn-Metal Anodes: Unique Challenges and Solutions

In this section, we deal with artificial SEI Layers for Zn-Metal anodes. Recently, polymer-based artificial SEIs have been reported to synergistically enhance zincophilicity, inhibit side reactions, and improve the long-term reversibility of Zn-metal anodes in aqueous batteries. These ultrathin polymer interphases leverage functional moieties (e.g., amide and carbonyl groups) that can coordinate with Zn^2+^ at the interface, creating zincophilic sites for facilitated Zn nucleation and growth [52]. For example, polyamide coatings rich in carbonyl and amine groups form robust Zn^2+^-coordination networks, guiding uniform, dendrite-free Zn deposition over >8000 h of cycling [52]. As a result, Zn anodes protected with polymer-based SEI layers exhibit greatly extended reversibility. Overall, such polymer-derived artificial SEIs provide zincophilic, water-repellent interphases that both coordinate Zn^2+^ and block deleterious species, thereby homogenizing Zn^2+^ transport and shielding the metal anode from hydrogen evolution (HER) and corrosion. This dual functionality has enabled markedly smoother Zn plating/stripping and longer cycling life in aqueous Zn batteries [53].

Compared with Li, metallic Zn in aqueous media encounters different challenges, including parasitic HER, interfacial corrosion/passivation, and texture-dependent dendritic or “mossy” growth that feeds on local pH and ion-flux gradients [54,55]. Therefore, the protective layer must tolerate hydration, buffer interfacial chemistry, and maintain water activity at the metal surface low enough to suppress the HER while also conducting Zn^2+^ and resisting fracture. One promising method for achieving this system is depositing fluorinated polymer interphases as ultrathin conformal films. Initiated chemical vapor deposition (iCVD) yields pinhole-free, sub-micrometer coatings (≈200 nm) with controlled elasticity and surface energy. On Zn foils, these layers lower the water activity at the interface, reduce the HER, and smooth nucleation, which together extend the symmetric-cell lifetime and enable pouch-cell demonstrations [56]. In addition, coatings that are too thin leave defects, while those that are too thick result in transport/polarization degradation. iCVD can be used to address these shortcomings [56].

**Figure 3:**
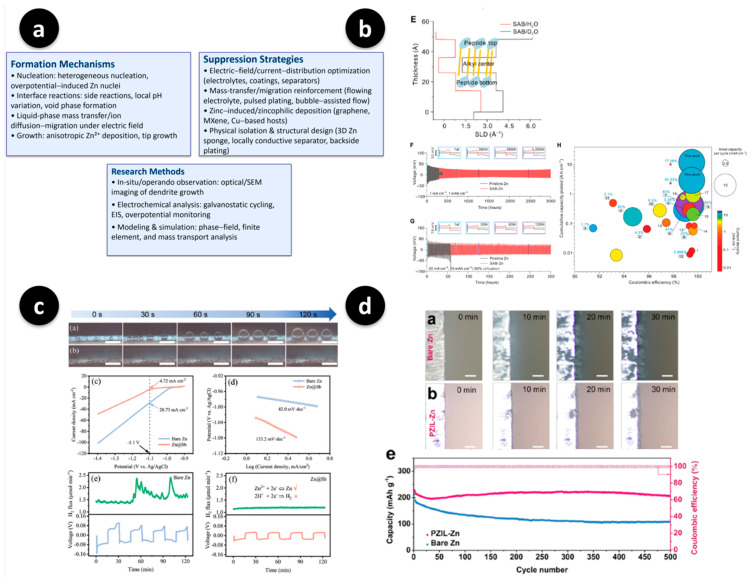
Polymer artificial SEIs for Zn metal anodes: mechanisms, remedies, and operando validation. (**a**) Redrawn schematic overview of Zn-dendrite formation mechanisms, research methods, and suppression strategies in aqueous systems, summarizing the design principles for interface engineering (source panel from Figure 2). Adapted and redrawn by the authors based on Zuo et al., *Materials Today Energy* (2021) [55]. (**b**) Enforced ordered planar plating/stripping under lean-electrolyte, high-areal-capacity conditions: neutron/reflectometry scattering length density (SLD) profile (E), long-term galvanostatic traces (F,G), and CE–areal-capacity benchmark bubble chart (H) evidencing planar growth stability. Source panels from Figure 1E and Figure 2F–H, adapted from Chen et al., *Science Advances*, 2024, 10(10), adn2265, under the terms of the Creative Commons Attribution NonCommercial License (CC BY-NC 4.0) [24]. (**c**) HER-suppression and interfacial kinetics with a zincophilic protective layer (Zn@Sb): time-lapse optical images of bubble evolution on bare-Zn vs. Zn@Sb (**a**,**b**), Tafel/kinetic analyses (**c**,**d**), and chronoamperometry/voltage oscillation readouts showing reduced H_2_ generation for Zn@Sb (**e**,**f**). Source panels from Figure 6a–f in Hong et al., *Advanced Science* (2022) [57]. (**d**) Zwitterionic bifunctional polymer interphase (poly zwitterionic ionic liquid (PZIL)-Zn). In situ optical microscopy images of symmetric cells showing smoother interfaces and delayed protrusion for PZIL-Zn compared with bare Zn (sub-panels (**a**,**b**); source panels from Figure 4a,b in Chen et al., *ACS Energy Letters* (2022) [16]). The full-cell durability at 1 C in terms of the capacity and Coulombic efficiency is also shown (sourced from Figure 5e in the same paper [16]. Reproduced/adapted from Ref. [16] with permission from the American Chemical Society. Note: Lower-case letters visible inside some sub-images correspond to the original figures’ sub-panel labels and are retained for traceability; scale bars and authors’ original annotations are preserved.

Figure 3 shows the key readouts for the Zn artificial SEIs. Figure 3a presents the aqueous Zn failure modes and mitigation strategies as a single design map, illustrating why coatings must address hydration, corrosion, and texture-driven growth [55]. Ordered planar plating/stripping under lean electrolyte and practical areal capacities are shown in Figure 3b, where the neutron/reflectometry SLD profile, long-term galvanostatic traces, and CE–capacity benchmark collectively indicate stable planar growth when the interphase is properly engineered [24]. Figure 3c shows the suppression of hydrogen evolution achieved using a zincophilic protective layer (Zn@Sb), as time-lapse images show fewer bubbles and smoother fronts, while the Tafel and chronoamperometry analyses quantify the reduced HER kinetics [57]. A zwitterionic bifunctional polymer interphase (PZIL-Zn) is presented in Figure 3d, wherein in situ symmetric-cell microscopy reveals a smoother interface than bare Zn, and full-cell cycling shows a higher capacity retention and Coulombic efficiency at 1 C [16]. Together, these data establish three practical rules for designing Zn artificial SEIs: ensuring a hydration-tolerant surface chemistry, conformal and defect-free coverage at sub-micrometer thicknesses, and operando-verified planar plating at realistic current densities and areal capacities.

A complementary strategy has been developed that uses zwitterionic polymers as the ion migration layers. Bifunctional zwitterionic coatings (or interphases derived from zwitterionic ionic-liquid polymers) promote selective Zn^2+^ transport while inhibiting water-driven side reactions; hydrogen evolution is measurably suppressed, and plating has been demonstrated to become increasingly uniform in long-term tests [16]. Besides zwitterions, polymer–inorganic hybrids and corrosion-resistant coatings are effective levers for planarizing deposition and reducing gas evolution [54]. Crucially, operando studies now focus on both the chemical effects of the coating and the resulting mechanical response of the anode. Using real-time probes, the ordered planar plating/stripping of Zn has been achieved by programming the interfacial bonding and energy landscape at the reaction front; the depth-of-discharge can be increased with minimal thickness fluctuation when planar growth is enforced [24]. At the same time, addressing the HER and dendrites together rather than in isolation has resulted in the largest stability gains [57].

In short, robust, aqueous Zn artificial SEIs require (i) hydration-tolerant chemistry (fluorinated or zwitterionic motifs); (ii) conformal, defect-poor coverage at sub-micrometer thicknesses to avoid transport penalties; and (iii) operando-verifiable planar growth under realistic rates and areal capacities. Although these principles are similar to those of Li systems, the controlling variables (pH, water activity, and corrosion equilibria) are Zn-specific and should be carefully designed [54,55].

## 6. Comparative Insights into Li- and Zn-Metal Polymer SEI Designs

The comparison summarized in Table 2 highlights that polymer SEI design principles diverge substantially between Li and Zn metal anodes. For Li systems, the key challenge lies in achieving mechanical reinforcement without compromising elasticity or ionic conductivity under highly reducing potentials. In contrast, Zn systems require chemically robust interphases capable of tolerating hydration and suppressing parasitic hydrogen evolution. These differences underscore that polymer chemistries optimized for Li cannot be directly transferred to Zn environments, explaining why a single universal design rule for polymer artificial SEIs remains unattainable.

## 7. Operando Visualization Techniques: From Ex Situ Analysis to Real-Time Investigation

For decades, the SEI has been visualized using ex situ analysis via depth profiling, XPS, FIB cross-sections, and post-mortem TEM. These methods establish the chemical constituents and layering of interphases; however, they cannot identify the growth, dissolution, and repair kinetics under a current [6]. While cells may appear compact after cycling, they may be highly dynamic during operation. To address these issues, electrochemical LC-STEM/TEM has been used to directly image metal plating and interphase rearrangement under bias, resolving nucleation statistics and tip growth in real-time [34,35]. Cryogenic electron microscopy (cryo-EM/ET) preserves beam- and vacuum-sensitive phases, enabling the visualization of nanocrystalline and amorphous motifs in SEIs close to their native states [21]. Spectroscopic techniques, including operando/in situ X-ray and vibrational probes (X-ray absorption spectroscopy (XAS)/XPS, Raman/infrared (IR) spectroscopy), can be used to determine the redox state, solvation motifs, and decomposition pathways as the interface evolves [21,25,34]. Together, these systems reveal the chemistry, morphology, and transport of these systems across timescales ranging from milliseconds to hours. Real-time readouts, including the nucleation overpotential, interfacial impedance drift, and roughness metrics from image statistics, can be obtained by adjusting the material parameters (i.e., polymer side chains, fluorination level, ceramic fraction, or cross-link density) to verify their effects on planar plating/stripping and failure. In aqueous Zn systems, for example, operando studies have demonstrated that programming interfacial bonding to favor lateral growth yields deep cycling with minimal thickness fluctuation [24], while in Li systems, liquid-cell/cryo-EM has clarified when “mosaic” or multiphase SEIs densify versus perforate under load [21,34].

These systems encounter two main issues. The first is related to the dose and environment. While the electron-beam chemistry and evaporative artifacts can bias liquid-cell experiments, cryo workflows mitigate but do not eliminate beam damage [21,35]. The second issue is related to the field of view. Nanometer-scale imaging should be complemented by area-averaged probes (e.g., operando synchrotron X-ray methods or impedance) to avoid the overinterpretation of rare events [58,59,60]. In the future, multimodal experiments that can perform imaging, spectroscopy, and electrochemistry in a single protocol are likely to transform operando tools from diagnostic systems into systems that inform the design of artificial SEIs.

**Figure 4:**
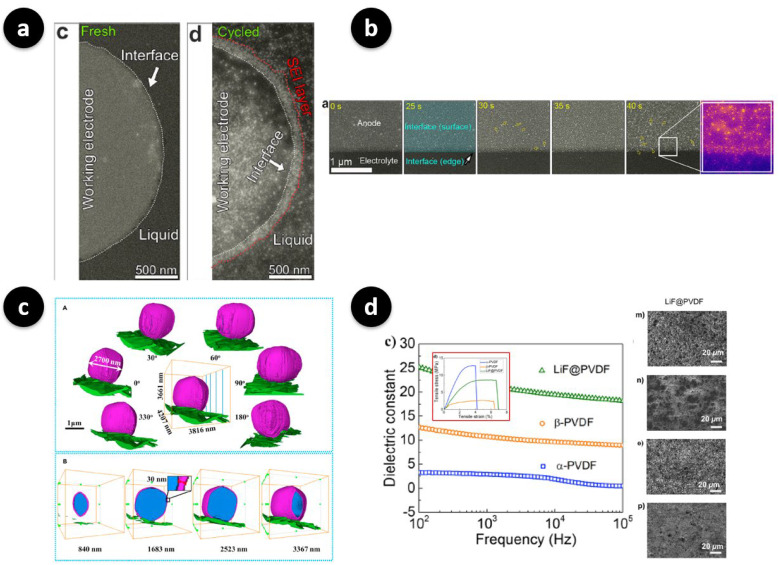
(**a**) Operando electrochemical LC-STEM (ADF-STEM) visualization of the nucleation and subsequent densification of a mosaic SEI at the lithiated interface, where the white arrows indicate the same smooth interface between the glassy-carbon (GC) electrode and the electrolyte before cycling, and the red dashed line marks the roughened SEI region formed after cycling (source panels from Figure 2c,d) [34] (Reproduced from Ref. [34] with permission from the American Chemical Society licensed under CC BY 4.0). (**b**) Time-resolved ADF-STEM images and corresponding electrochemical profiles illustrating the nucleation, island-like growth, and morphological evolution of the SEI layer on the GC electrode during cycling (source panel from Figure 3a) [34]. (Reproduced from Ref. [34] with permission from the American Chemical Society licensed under CC BY 4.0). (**c**) Low-dose cryogenic electron-microscopy tomography of Li metal anodes: colored 3D reconstructions of a Li deposit (blue) conformally coated by an SEI (purple) on Cu (green), together with depth-resolved slices that enable quantitative extraction of particle size (≈2.7 µm) and SEI thickness (≈30 nm). Source panels from Figure 3A,B in Li et al., iScience (2021) [23]. Reproduced from Ref. [23] under the Creative Commons CC BY license. (**d**) High-dielectric PVDF/LiF artificial SEI promotes uniform plating at a high areal capacity, as confirmed by SEM and dielectric metrics [14] (Adapted from Ref. [14] with permission from the American Chemical Society). Note: Lower-case letters visible inside some sub-images correspond to the original figures’ sub-panel labels and are retained for traceability; scale bars and authors’ original annotations are preserved.

As concrete illustrations of what these methods can uncover, Figure 4 brings together four complementary perspectives. Panel 4a presents operando electrochemical LC-STEM imaging of a lithiated interface, where a mosaic SEI forms and subsequently densifies under applied bias, directly visualizing the interphase rearrangement rather than relying on post-mortem contrast [34]. Panel 4b shows operando LC-STEM observations of SEI nucleation and growth on a GC electrode, revealing the real-time morphological evolution of the SEI layer during electrochemical cycling [34]. Panel 4c highlights the unique contribution of cryo-EM and tomography, where three-dimensional reconstructions reveal Li deposits conformally coated by SEI layers, while depth-resolved slices provide quantitative information such as particle size and SEI thickness in the tens-of-nanometers regime, all without destroying beam-sensitive phases [23]. Finally, panel 4d closes the loop between observation and mitigation, showing that a high-dielectric PVDF/LiF artificial SEI produces smoother plan-view morphology and a dielectric response indicative of more uniform plating at practical areal loadings, which illustrates how operando-derived metrics can directly inform polymer design decisions [14]. Taken together, these snapshots explain why combining imaging, spectroscopy, and standard electrochemical measurements transforms SEI visualization from a qualitative exercise into a feedback tool for interface engineering.

The significance of this coupling is practical rather than merely descriptive. Real-time metrics such as nucleation overpotential, interfacial impedance drift, and statistical roughness parameters extracted from images can be systematically aligned with key design variables, including polymer side-chain chemistry, fluorination level, ceramic fraction, or cross-link density, to reveal which design choices genuinely enforce planar plating and stripping and which simply relocate failure elsewhere. For example, in aqueous Zn systems, operando studies have shown that tuning interfacial bonding to promote lateral growth leads to deep cycling with minimal thickness fluctuations [24], while in Li systems, liquid-cell and cryo-EM have clarified when mosaic or multiphase SEIs densify versus when they perforate under load [21,34]. Two caveats are critical when interpreting such data. First, beam dose and environmental effects can influence the results because electron-beam chemistry and evaporative artifacts can distort liquid-cell measurements, while cryo workflows mitigate but do not eliminate beam damage [21,35]. Second, the field of view needs to be considered carefully since nanoscale imaging should be complemented by area-averaged probes, such as operando synchrotron X-ray methods or impedance spectroscopy, to avoid over-emphasizing rare local events [58,59,60]. Looking ahead, multimodal experiments that co-register imaging, spectroscopy, and electrochemistry within a single protocol are likely to transform operando techniques from diagnostic tools into design engines for artificial SEIs.

## 8. Outlook and Future Perspectives: Toward Rational Design and Application

The realization of polymer-based artificial SEI layers in Li- and Zn-metal anodes is increasingly being achieved by coupling synthesis and operando analyses with modeling. In practice, this includes the design of the polymer architecture (ionomer content, cross-linking density, and ceramic fraction), prediction of its transport/mechanics, and immediate verification of those predictions with real-time readouts (nucleation overpotential, interfacial impedance drift, and roughness statistics) before the next chemical iteration [34,42]. Such systems have been applied to other battery types, such as data-driven or Bayesian optimization, which propose design conditions, measure outcomes, and update the model parameters [61,62]. LC/cryo-EM and operando X-ray/vibrational probes can be used to aid in interphase design [21,35]. Polymer-based artificial SEIs should be designed to achieve a high Li^+^/Zn^2+^ transference, intermediate modulus with good wetting/adhesion, high electron resistivity, and chemical tolerance to the chosen electrolyte. In addition, these design parameters should be identifiable via an operando analysis. For example, the fluorination level and ionomer fraction relate to the interfacial water activity/HER for Zn, while the cross-link chemistry and filler loading relate to planar plating and stack swelling for Li [24,55]. Failure may also be identified using this method, as when inactive Li accumulates or the system thickness periodically expands and contracts, the design and test protocol must be revised [48]. Next-generation interphases must be designed considering their intended use rather than coin-cell benchmarks, and their design may include the use of lean electrolytes and a limited N/P ratio, high areal capacity and current, and pouch-cell stack pressure uniformity [49,50,51]. Therefore, polymer-based artificial SEIs should be designed in conjunction with liquid electrolytes in LMBs, considering the importance of their transport and adhesion characteristics.

**Figure 5:**
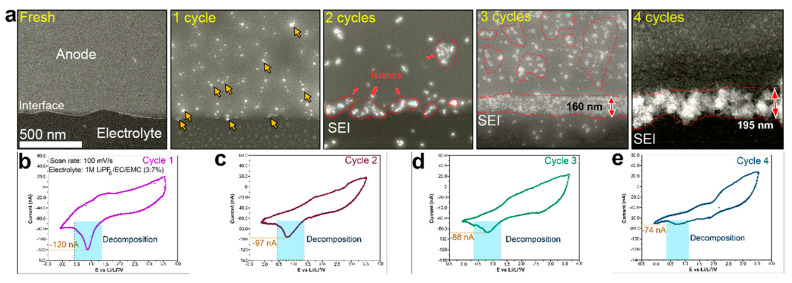
Evolution of the SEI layer with an increasing number of cycles: (**a**) time series of ADF-STEM images showing the evolution of the SEI at the GC electrode, yellow arrows indicate the initial nucleation sites of SEI formation during the first cycle, and red dashed lines mark the boundary of the growing SEI layer with increasing cycles. (**b**–**e**) first, second, third, and fourth CVs, respectively, obtained from an operando ethylene Carbonate (EC) LC-STEM experiment showing the charge–discharge characteristics of the GC electrode in a LiPF6/EC/EMC liquid electrolyte at a flow rate of 2.5 μL/min, where the colored curves denote different cycle numbers and the blue-shaded regions indicate the potential range of electrolyte decomposition. Source panels from Figure 4, reproduced in part from Ref. [34] with permission from the American Chemical Society under CC-BY 4.0 license).

In a previous study [34], operando electrochemical LC-STEM was used to observe the formation and evolution of an SEI on a graphitic anode in a lithium-ion battery. It was found that SEI formation was not a single-step process, as it began at a relatively high potential and proceeded via multiple stages, as shown in Figure 5.

This review identified several important parameters affecting polymer-based artificial SEI layers in Li- and Zn-metal anodes. While aqueous Zn is governed by pH and corrosion equilibria, sodium differs in its solvation and interfacial mechanics, and lithium is the most reductive among these metals and exposes nanoscale defects rapidly. Therefore, a universal optimal SEI is unlikely to be achieved, and an adaptable framework considering tunable ionomers or block-copolymers, dynamic bonds for self-repair, and graded polymer–ceramic hybrids can be optimized considering operando metrics for each system [47,54]. Therefore, data-informed, dynamically validated interface designs should be developed.

**Figure 6:**
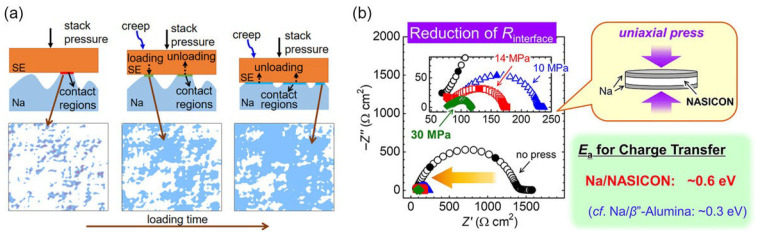
(**a**) 3D time-dependent model describing the interface evolution between Na metal and Na-β″-Al_2_O_3_ under stack pressure, and (**b**) schematic illustration of Na^+^ ion transfer at the Na/NASICON interface under uniaxial compression; adapted from Lu et al., *Energy Technol*. (2022) [47] ©Wiley-VCH GmbH, published under the CC BY-NC-ND license, with panel (**a**) reprinted with permission from Zhang et al., ACS Appl. Mater. Interfaces (2021) [63] ©American Chemical Society, and panel (**b**) reprinted with permission from Uchida et al., ACS Appl. Energy Mater. (2019) [64] © American Chemical Society.

A previous review [47], as shown in Figure 6, noted that no single strategy is sufficient for optimizing polymer-based artificial SEI layers in Li- and Zn-metal anodes. Improved performances are often obtained when multiple strategies are combined (e.g., a sodiophilic scaffold + protective coating + electrolyte additives). However, there is still a tradeoff between stability, safety, and performance (i.e., conductivity and rate capability) in these systems.

**Figure 7:**
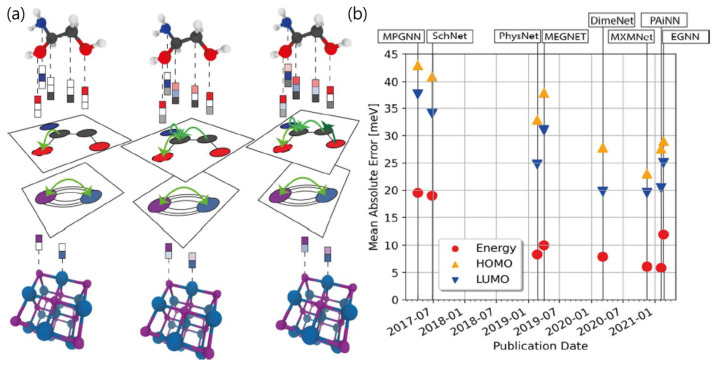
Overview of message-passing–based GNNs for molecules and crystals. (**a**) Message passing in which atomic/node features are updated by exchanging information along chemical bonds or interatomic connections. (**b**) QM9 benchmark: mean absolute errors for total/internal energy (red circles), HOMO level (orange triangles), and LUMO level (blue inverted triangles) reported for representative GNN models published between 2017 and 2021. Adapted from Reiser et al. [67] under the Creative Commons Attribution 4.0 license.

Machine learning has been increasingly applied to materials science applications, including SEI design. A recent review [65] highlighted how machine learning (ML) is revolutionizing materials research by accelerating discovery, optimizing properties, and achieving predictive modeling. A recent survey comprehensively reviewed the rapid growth of materials machine learning by analyzing commonly used software, databases, and algorithms, highlighting that conventional ML methods still dominate over deep learning in most materials problems, and identifying critical challenges related to data size, extrapolation, interpretability, and access. These insights are particularly relevant for polymer-based artificial SEI research, where data scarcity and complex interfacial chemistries often necessitate careful selection of appropriate ML models and feature representations [66]. Another study [67] focused on using graph neural networks (GNNs) for molecular and material property predictions. GNNs are a key ML method that builds upon the ideas of Butler et al. regarding representation and structure. In a recent work [68], machine-learning interatomic potentials (MLIPs) (i.e., moment tensor and GNN potentials) were used to model SEI components, including Li_2_CO_3_ and Li_2_EDC, allowing large-scale structure and dynamics simulations for mixed materials. This framework could be extended to polymer/artificial SEIs by training MLIPs on polymer–inorganic composite interfaces to capture nanoscale deformation, Li^+^ transport heterogeneity, and interfacial stress accumulation during cycling. Beyond atomistic simulations, ML models can also incorporate polymer-specific structural descriptors, such as monomer sequence, co-monomer ratio, chain length, cross-link density, and filler loading fraction, as input features to predict operando metrics including interfacial adhesion, elastic recovery, and dendrite penetration probability. For instance, GNN-based models could represent polymer–filler networks as graphs where nodes correspond to monomer segments or inorganic clusters and edges encode mechanical or ionic interactions, thereby learning how mesoscale topology governs interfacial durability. Similarly, MLIPs or Gaussian-process regressors can be embedded in closed-loop frameworks combining ML prediction, molecular simulation, and targeted experimentation to iteratively optimize polymer composition and cross-linking degree for maximum SEI stability. Another recent review by Sun et al. [69] examined the application of ML techniques in combination with simulations and machine vision to predict SEI formation and dynamic interfacial behaviors. In particular, Sun et al. [69] highlighted how ML-assisted simulations can capture dendrite growth, SEI evolution, and interfacial transport phenomena, offering predictive insights that complement traditional experimental methods. Building on these developments, polymer artificial SEI research can adopt similar ML-driven, feedback-optimized pipelines to design adaptive coatings that reconcile mechanical robustness with interfacial elasticity. These concepts are illustrated in Figure 7, which highlights representative ML-based strategies for modeling battery interfaces. Such approaches can also be extended to the design of artificial polymer-based SEIs.

## 9. Conclusions

Polymer-based artificial SEIs are increasingly being designed using data-based approaches. In these systems, the molecular design is determined using operando observables, such as the nucleation overpotential, interfacial impedance drift, and roughness statistics, to stabilize metal deposition across Li and Zn systems [21,34,42]. In these systems, the coatings must function under realistic constraints, including lean electrolyte, limited N/P, high areal capacity/current density, and uniform stack pressure conditions, and they should be able to undergo scale-up from coin to pouch cells with minimal performance degradation [49,50,51]. To achieve this, a closed-loop design system is required in which parameters such as the ionomer/fluorination level, cross-linking, and polymer–ceramic fraction are quantitatively tied to operando metrics and revised when failure modes, such as inactive Li buildup or stack swelling, appear [48]. In aqueous Zn systems, the pH and aqueous activity dictate the corrosion/HER and growth modes, and enforcing planar plating/stripping with hydration-tolerant interphases can be used to achieve deep-cycling behavior [24,54,55]. In Li systems, liquid-cell/cryo-EM studies have connected SEI composition and mechanics to morphology analyses conducted in real time, providing feedback required to realize durable coatings [21,34].

These findings indicate that identifying the appropriate parameters for optimization, measuring characteristics associated with these parameters in real-time, and iterating this process may be used to optimize polymer-based artificial SEIs in Li- and Zn-metal batteries. Prototypes of these batteries may be designed and fabricated using this workflow.

## Figures and Tables

**Table 1:** Mapping of polymer artificial SEI design parameters to their target interfacial functions and the corresponding operando/diagnostic observables. Representative literature supporting each mapping is indicated.

Design Parameters	Target Interfacial Function	Operando/Diagnostic Observable	Representative Evidence
Zwitterionic/ionomer side-chains	Cation-selective transport; suppressed solvent co-transport	Planar plating in LC-TEM; lower *R_ct_* drift (operando EIS)	[39,41]
Fluorinated/salt-philic side-chains	Inorganic-rich, electronically insulating SEI (e.g., LiF-rich)	Denser mosaic SEI; suppressed porosity growth (operando LC-STEM/X-ray)	[34,37]
Ceramic fillers (Al_2_O_3_, LLZO) in polymer	Raised modulus; homogeneous current distribution	Reduced tip-growth probability; smoother front in LC-TEM	[35,38,40]
Dynamic cross-links/supramolecular bonding	Self-healing of microcracks; coverage retention	Stable plating morphology under cycling; slower impedance rise	[36]

Abbreviations: LC-TEM/LC-STEM, liquid-cell transmission/scanning transmission electron microscopy; *R_ct_*, charge-transfer resistance; SEI, solid electrolyte interphase; LLZO, Li_7_La_3_Zr_2_O_12_.

**Table 2:** Comparative design insights for polymer-based artificial SEI layers on Li- and Zn-metal anodes.

Aspect	Li-Metal Anodes	Zn-Metal Anodes
Operating environment and failure modes	Operates in non-aqueous carbonate or ether electrolytes under a highly reducing potential (−3.04 V vs. SHE). Unstable inorganic SEI formation leads to electron-driven dendritic growth and accumulation of inactive lithium.	Functions in aqueous or mildly alkaline electrolytes (−0.76 V vs. SHE). Corrosion, hydrogen evolution, and ion-depletion-driven mossy growth are the primary degradation pathways.
Mechanical and interfacial stress	Large volume fluctuation (>10%) during cycling requires a stiff yet elastic SEI to prevent cracking and delamination.	Moderate volume change but strong hydration-induced swelling demands cohesive and hydrophobic polymer coatings.
Design priorities	High modulus (>1 GPa) and fracture toughness to suppress dendrite penetration while maintaining electronic insulation and interfacial adhesion.	Hydration resistance, zincophilicity for homogeneous Zn^2+^ flux, and inhibition of hydrogen evolution and corrosion.
Representative polymer design	PVDF-HFP, PAN, and PEO-based copolymers, often combined with LiF or Li_3_N fillers to enhance mechanical robustness and ion selectivity.	Polyamide, polyacrylate, PVA, chitosan, and zwitterionic copolymers containing Zn-coordinating amide or hydroxyl groups.
Targeted interfacial function	Uniform Li^+^ transport, suppression of filament nucleation, and self-healing adhesion at the Li–polymer interface.	Zincophilic coordination networks ensuring uniform Zn^2+^ transport, reduced hydration, and hydrophobic shielding against HER.
Operando readouts and key metrics	Ionic conductivity (*σ*), Li^+^ transference number (*t^+^*), charge-transfer resistance (*R_ct_* or *R_SEI_*), nucleation overpotential (*η_nuc_*), and modulus (*E*/*H*/*G_c_*). LC-TEM and EIS reveal crack arrest and filament deflection in reinforced polymers.	Coulombic efficiency (CE), *R_ct_*, corrosion current density, and hydrogen-evolution rate (HER). Optical and neutron reflectometry demonstrate planar Zn plating and bubble suppression.
Representative examples	Wang et al. reported a LAGP–PVDF-HFP hybrid SEI exhibiting a modulus of 25 GPa and uniform Li deposition [30].	Youk et al. demonstrated LiPAA and PDMS coatings achieving dendrite-free Zn cycling for over 8000 h [31].
Design implication	The Li system demands a delicate balance between rigidity and elasticity to accommodate extreme reduction and mechanical stress.	The Zn system relies on hydrophobicity and Zn-affinity to stabilize aqueous interfaces and mitigate HER-driven degradation.

## Data Availability

No new data were created or analyzed in this study. Data sharing is not applicable to this article as all data discussed are available from previously published sources cited within the manuscript.
